# God Help Her

**Published:** 1892-09

**Authors:** James McCarroll


### GOD HELP HER.

God help the wretch who nightly drags
Her life against the ghastly flags,
In sin, in hunger, and in rags.

God help her when the bitter rain
Beats on her—like a window pane—
And almost washes out her stain.

God help her when, with bleeding feet,
She pauses ere she stoops to meet
The cruel corner of the street.

God help her when, with tearless eye,
She looks into the blackened sky,
And strikes her breast and asks to die.

God held her, wandering to and fro
Without one pitying look to throw
A gleam upon her sullied brow.

Poor child of good, and child of ill,
The slave of her misguided will ;
God help her !—She's a woman still !

JAMES McCARROLL, *in Health and Humanity.*

